# Dynamics of Th9 cells and their potential role in immunopathogenesis of murine schistosomiasis

**DOI:** 10.1186/s13071-017-2242-1

**Published:** 2017-06-24

**Authors:** Tingzheng Zhan, Tingting Zhang, Yanyan Wang, Xiaoli Wang, Cai Lin, Huihui Ma, Zhongliang Duan, Chunxiang Li, Jing Xu, Chaoming Xia

**Affiliations:** 1 Department of Parasitology, Medical College of Soochow University, 199 Renai Road, Suzhou, 215123 China; 2 Department of Parasitology, Guangxi Medical University, 22 Shuangyong Road, Nanning, 530021 China; 3 Department of Parasitology, Bengbu Medical College, 2600 Donghai Road, Bengbu, 233030 China

**Keywords:** Th9 cell, Interleukin-9, PU.1, Schistosomiasis, Egg granuloma

## Abstract

**Background:**

Th1, Th2, Th17, Treg and Tfh cells play important roles in schistosomiasis. Th9 cells secrete IL-9 as a signature cytokine and contribute to several classes of inflammatory disease. However, the effects of Th9 cells in schistosomiasis are unknown. We aimed to explore the dynamic changes and potential roles of Th9 cells in the pathogenesis of hepatic egg granulomatous inflammation in mice infected with *Schistosoma japonicum*.

**Methods:**

Twenty mice with *S. japonicum* infection and five normal controls (NC) were used as models. The average areas of egg granulomas were estimated by hematoxylin-eosin (H & E) staining. Hepatic IL-9 and transcription factor PU.1 levels were detected by immunohistochemistry. Flow cytometry techniques were used to analyze the proportions of Th9 cells. With the help of ELISA, serum levels of IL-9 were examined.

**Results:**

The egg granulomas began to form from four weeks after infection and continued to develop. In parallel with the development of egg granulomas, the hepatic levels of IL-9 and PU.1 increased very slowly during the first four weeks post-infection and increased rapidly thereafter. Moreover, the proportions of splenic Th9 cells and levels of serum IL-9 had similar developmental trends with the egg granulomas.

**Conclusion:**

The proliferation of Th9 cells and levels of IL-9 were significantly higher in *S. japonicum*-infected mice compared to NC. In addition, dynamic changes of Th9 and IL-9 were synchronous with the developmental trend of hepatic egg granulomatous inflammation, suggesting that Th9 cells might be a new subset in the pathogenesis of schistosomiasis.

## Background

Schistosomiasis is a major neglected helminthic disease infecting approximately 200 million people and threatening close to 700 million people; it is caused by *Schistosoma* spp. that are widely distributed in more than 70 countries in tropical and subtropical regions [1]. With such alarming statistics schistosomiasis remains one of the most serious public problems. Of the five known species of schistosomes infecting humans, *S. japonicum* is an important parasite in China. People can become infected by contacting infested water containing *S. japonicum* cercariae. Cercariae invade human body through the skin and become schistosomules. Schistosomules migrate to the mesenteric veins where they grow into adult worms [2]. The main immunopathology during infection with *S. japonicum* develops as a result of schistosome eggs that lodge in host liver and intestines causing extensive tissue damage. Much of the symptomatology of schistosomiasis is contributed to the egg-induced granulomatous inflammation and secondary fibrosis [1]. However, the mechanism underlying the development of this pathological change is still insufficiently studied.

CD4^+^ T cells are essential for both host immune responses against schistosomes and immunopathology in schistosomiasis. Especially granulomatous and fibrosing inflammation are totally orchestrated and facilitated by CD4^+^ T cells [3]. CD4^+^ T-cell-deficient mice fail to establish an effective granulomatous response. Upon the stimulation of schistosome antigens, naive CD4^+^ T cells differentiate into distinct effector subsets of T-helper (Th). Up to now, five subsets are found to be involved in the pathogenesis of schistosomiasis, including Th1, Th2, Th17, T regulatory (Treg) and T follicular helper (Tfh) cells. Th2, Th17 and Tfh cells could upregulate hepatic granuloma formation via secreting IL-4, IL-17 and IL-21, respectively [4–9], while Th1 and Treg cells play an opposite role by producing IFN-γ and IL-10, respectively [10–13].

Th9 has recently been described as a unique subset of CD4^+^ T cells that is named because of the signature cytokine IL-9 secreted after activation. And PU.1 and IRF-4 are specific transcription factors of Th9 cells [14–16]. IL-9 used to be thought a Th2-specific cytokine more than 20 years ago. Until 2008 two papers reported that IL-9 could be secreted exclusively a new subpopulation of CD4^+^ T cells termed Th9 cells [17, 18]. To date, Th9 cells appear to have function in a broad range of autoimmune disorders, allergic inflammation and cancers as well as the parasitic infection [14, 19, 20]. However, whether Th9 cells, as the other CD4^+^ T cell subsets do, are involved in the liver pathology induced by *S. japonicum* infection has never been explored. Therefore, we aim to investigate the relation between the dynamic changes of Th9 cells and hepatic egg granulomatous inflammation in *S. japonicum*-infected mice. This study may provide novel insights regarding the potential role of Th9 cells in immunopathogenesis of schistosomiasis.

## Methods

### Mice, parasites and infection

Female ICR mice at 6–8 weeks of age with body weights of 18–20 g were purchased from SLAC Laboratory (Shanghai, China). All mice were housed under specific pathogen-free conditions (12 h light/12 h dark; temperature, 22–24 °C) at the laboratory animal research facility of Soochow University (Suzhou, China). *Oncomelania hupensis* harboring *S. japonicum* cercariae (Chinese mainland strain) were purchased from Jiangsu Institute for Schistosomiasis Control (Wuxi, China). Twenty-five mice were randomly divided into experimental group (*n* = 20) and normal control group (*n* = 5). In the experimental group, each mouse was infected with 15 ± 1 cercariae of *S. japonicum* through the abdominal skin, and five mice were chosen randomly in experimental group at 4, 7, 9 and 12 weeks post-infection (pi) and euthanized for further studies.

### Histopathological study

The harvested liver specimens were fixed in 10% buffered formalin, embedded in paraffin blocks and cut into 4 μm thick serial sections. Liver sections were stained with hematoxylin and eosin (H & E) for granulomas analysis. The diameters of granulomas (25/mouse) surrounding single eggs were measured using an ocular micrometer, and the area of each granuloma was calculated assuming a circular shape.

### Immunohistochemistry

Liver sections were deparaffinized in xylene and rehydrated in alcohol and distilled water. Immunostaining for IL-9 and PU.1 were performed using monoclonal anti-IL9 primary antibody (Abcam, Cambridge, UK) and monoclonal anti-PU.1 primary antibody (Cell Signaling Technology, Danvers, MA, USA), respectively. The immunohistochemical technique used a two-step method (peroxidase-conjugated polymer). Antigen retrieval was performed by pressuring heating sections for 8 min in citrate buffer (pH 6.0). Three percent H_2_O_2_ was used to block the endogenous peroxidase activity, and the non-specific binding was eliminated with 10% normal goat serum in 0.01 M PBS for 30 min at room temperature. All sections were incubated free-floating with primary antibodies at 37 °C for 1 h and then at 4 °C overnight. The sections were further incubated with the secondary antibodies (ChemMate Envision/HRP, rabbit/mouse detection IHC kit, Gene-tech, Shanghai, China) at 37 °C for 1 h. Immunoreactivity was visualized using diaminobenzidine (DAB) as the chromogen. Five high-power fields (×400) per mouse liver were randomly photographed with a light microscope (Olympus, Tokyo, Japan). The integrated optical density (IOD) of IL-9-positive cells and PU.1-positive cells were measured by Image-Pro Plus 7.0 software (Media Cybernetics, Bethesda, MD, USA).

### Flow cytometry

Single-cell splenocyte suspensions were prepared by mincing the spleens in PBS containing 1% FBS (Gibco, Grand Island, NY, USA). Red blood cells were lysed using ACK lysis buffer. Approximately 10^6^ splenocytes in 100 μl were stimulated with 50 ng/ml PMA (Sigma-Aldrich, St. Louis, MO, USA) and 1 μg/ml ionomycin (Sigma-Aldrich) in the presence of 10 μg/ml Brefeldin A (BD Biosciences, San Jose, CA, USA) for 4 h at 37 °C in 5% CO_2_. After 5 h incubation, the cells were surface stained with FITC anti-mouse CD4 antibody (BD Bioscience PharMingen, San Jose, CA, USA) at 4 °C for 30 min in the dark. Subsequently, the cells were fixed and permeabilized with Cytofix/Cytoperm buffer (BD Biosciences) and then intracellularly stained with PerCP-Cy5.5 conjugated anti-mouse IL-9 (BD Bioscience PharMingen). All of the above procedures were performed on ice until the time of analysis. All of the stained cells were evaluated using a Flow Cytometer (Beckman Coulter, Brea, CA, USA) and data were analyzed with CXP software.

### Enzyme-linked immunosorbent assay (ELISA)

The concentrations of IL-9, IL-4 and TGF-β in serum were measured by ELISA Ready-SET-Go kits (Multi sciences, Hangzhou, China) according to the manufacturer’s instructions. The optical density (OD) of the plates was read at 450 nm using an ELISA reader and the concentrations were calculated using standard curves.

### Statistical analysis

All statistical analyses were carried out with SPSS21.0 Data Editor (SPSS Inc., Chicago, IL, USA). Data were shown as the mean ± standard deviation (SD). Comparisons of the same parameters in multiple datasets or more than two groups was performed using one-way analysis of variance (ANOVA). Differences were considered statistically significant when *P* < 0.05 or *P* < 0.01.

## Results

### The area changes of hepatic egg granulomas during infection

**Fig. 1:**
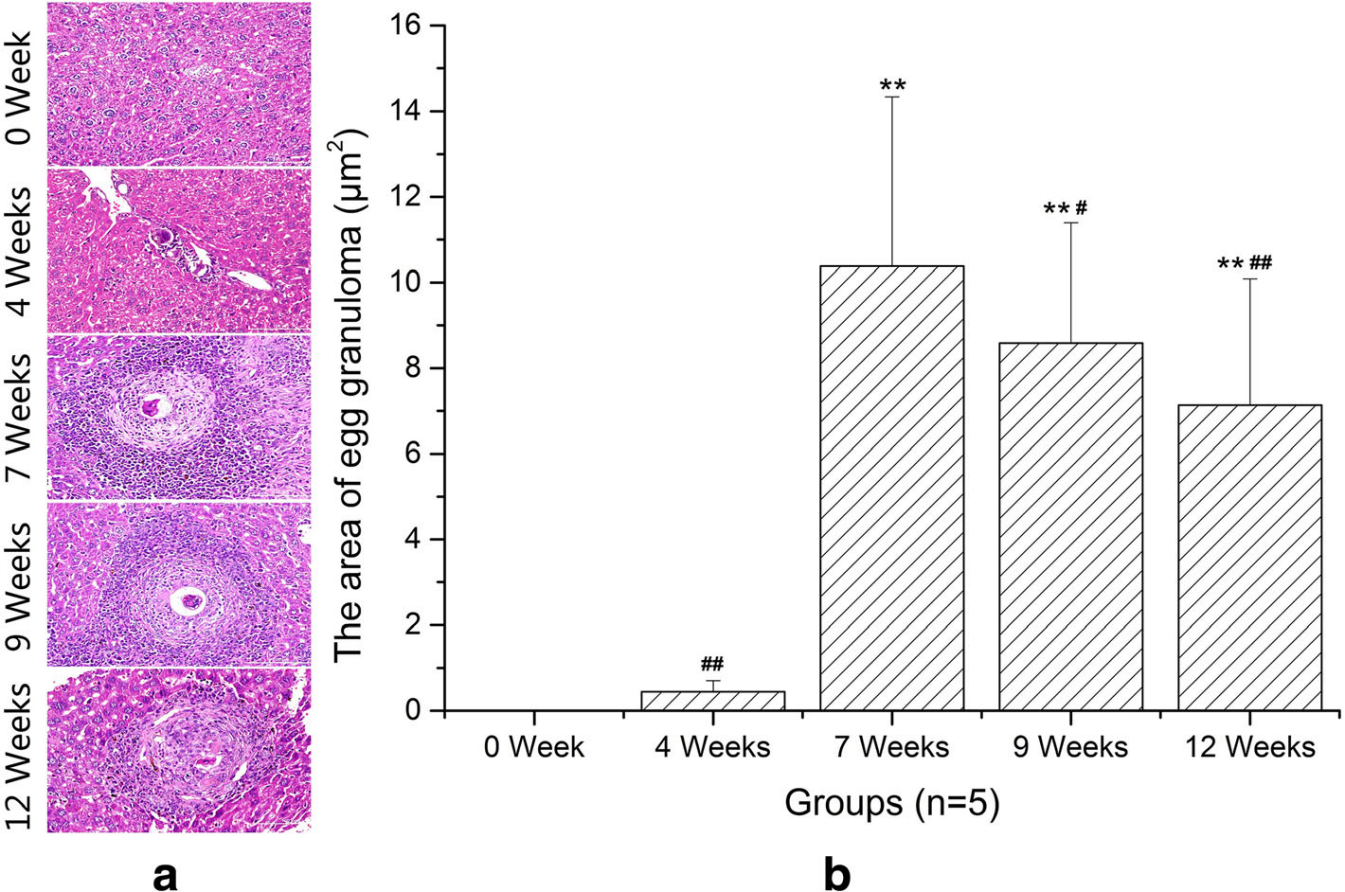
Histology of liver sections stained with hematoxylin-eosin staining. **a** Representative graphs of hepatic egg granuloma from *S. japonicum*-infected mice at 0 (NC), 4, 7, 9 and 12 weeks (original magnification: ×400). **b** Comparison of the areas of hepatic egg granuloma among different time groups. Data are presented as mean ± SD. **P < 0.01 compared with NC; ^#^ *P* < 0.05, ^##^ *P* < 0.01 compared with 7 weeks. *Scale-bars*: 100 μm

H & E staining showed that liver-trapped eggs became focal points for inflammatory infiltrates and obvious granulomas were observed under optical microscopy from 7 weeks after *S. japonicum* infection (Fig. 1a). As shown in Fig. 1b, hepatic egg granulomatous inflammation was the most serious at 7 weeks, and then granulomas shrank gradually (ANOVA: *F*_(4,20)_ = 86.76, *P* < 0.0001).

### The expression changes of IL-9 in the hepatic granulomatous inflammation formation during infection

**Fig. 2:**
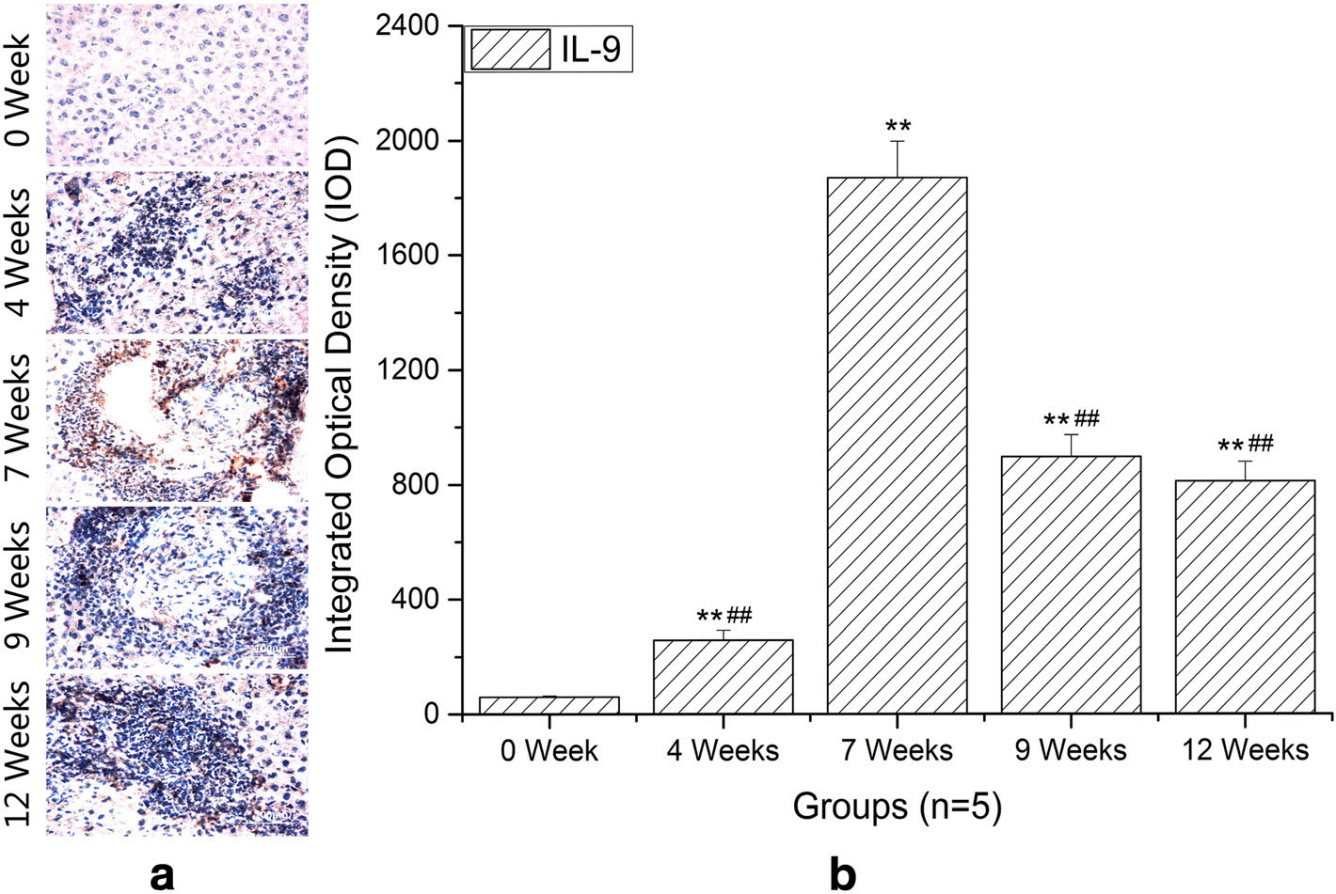
Immunohistochemistry of IL-9 in livers. IL-9^+^ cells localized mainly in the granulomatous areas. **a** Representative graphs of hepatic expression and distribution of IL-9 from *S. japonicum*–infected mice at 0 (NC), 4, 7, 9 and 12 weeks (original magnification: ×400). **b** Comparison of the IOD of IL-9 among different time groups. Data are presented as mean ± SD. **P < 0.01 compared with NC; ^##^ *P* < 0.01 compared with 7 weeks. *Scale-bars*: 100 μm

IL-9 is the typical cytokine produced by Th9 cells and plays a potent proinflammatory role. As shown in Fig. 2b, the liver IL-9 expression was significantly higher in *S. japonicum*-infected mice compared with normal controls (ANOVA: *F*_(4,20)_ = 693.20, *P* < 0.0001) and accumulated mainly in the areas of granulomatous response surrounding a parasite egg (Fig. 2a). In addition, the expression of IL-9 reached a peak at 7 weeks and then descended. Thus it can be seen that the level of hepatic IL-9 was correlated with hepatic granulomatous inflammation.

### The expression changes of PU.1 in the hepatic granulomatous inflammation formation during infection

**Fig. 3:**
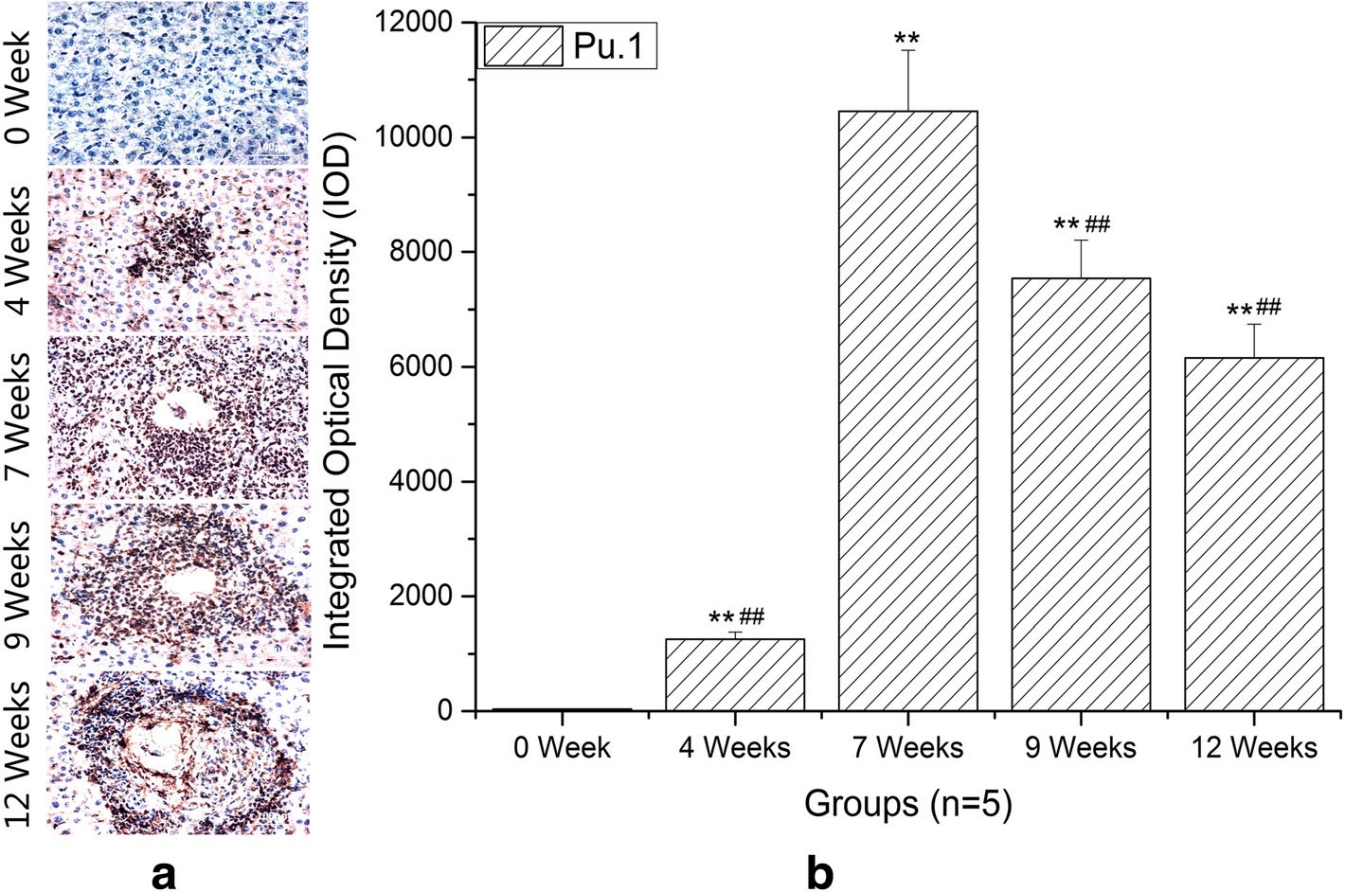
Immunohistochemistry of PU.1 in livers. **a** Representative graphs of hepatic expression and distribution of PU.1 from *S. japonicum*-infected mice at 0 (NC), 4, 7, 9 and 12 weeks (original magnification: ×400). **b** Comparison of the IOD of PU.1 among different time groups. Data are presented as mean ± SD. **P < 0.01 compared with NC; ^##^ *P* < 0.01 compared with 7 weeks. *Scale-bars*: 100 μm

The differentiation of Th9 cells depends on specific transcription factor PU.1. To confirm the distribution and frequency of Th9 cells in the liver, we investigated the IOD of PU.1 by immunohistochemistry. As expected, expression of PU.1 was dramatically increased in the liver of *S. japonicum*-infected mice compared to normal controls (ANOVA: *F*_(4,20)_ = 476.08, *P* < 0.0001) (Fig. 3b) and localized mainly in the areas of granulomatous response surrounding a parasite egg (Fig. 3a). Moreover, the trend of PU.1 expression was synchronous with IL-9 expression.

### The proportion changes of splenic Th9 cells at different infection stages

**Fig. 4:**
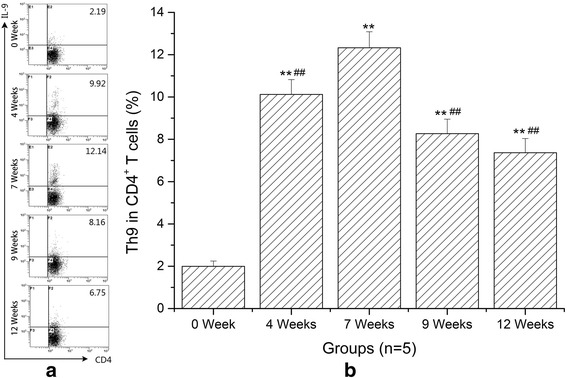
Percentages of Th9 cells in total CD4^+^ T cells from spleens significantly increased in mice with schistosomiasis. **a** Representative dot plots of Th9 cells by flow cytometry in *S. japonicum*-infected mice at 0 (NC), 4, 7, 9 and 12 weeks. All of the values were gated on CD4^+^ cells. The percentages of Th9 cells in CD4^+^ cells are indicated in the upper right of each chart. **b** Quantitative changes of Th9 cells in splenic CD4^+^ T cells at different time points. Data are presented as mean ± SD. **P < 0.01 compared with NC; ^##^ *P* < 0.01 compared with 7 weeks

In this study, to clarify the dynamic changes of splenic Th9 cells in *S. japonicum*-infected mice, CD4^+^ IL-9^+^ T cells were defined as Th9 cells. As shown in Fig. 4a, the proportions of Th9 cells in splenic CD4^+^ T cells were detected by flow cytometry. The results showed that the proportions of Th9 in CD4^+^ T cells significantly elevated as the infection developed than in normal controls (ANOVA: *F*_(4,20)_ = 183.44, *P* < 0.0001) (Fig. 4b). In addition, the proportion of Th9 increased very rapidly at 4 weeks and peaked at 7 weeks. Subsequently, the number of Th9 cells declined gradually. However, the level of IL-9 in liver increased very slowly at 4 weeks post-infection compared to that in spleen. The reason for this is that eggs begin to deposit in the mouse liver from 4 weeks after infection and then granulomatous inflammation forms gradually [6].

### The concentration changes of IL-9 in serum at different infection stages

**Fig. 5:**
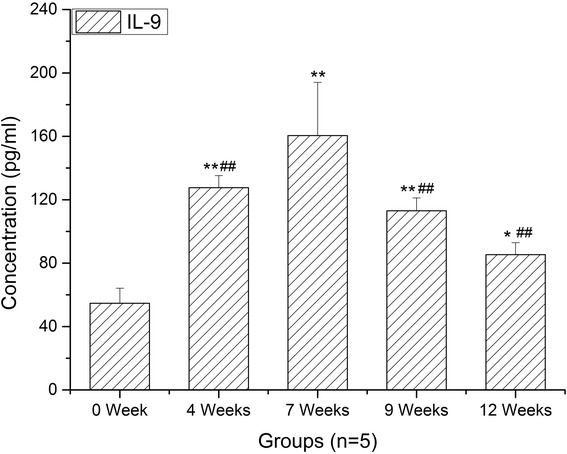
Kinetics of serum IL-9. Enzyme-linked immunosorbent assays (ELISA) was used to measured IL-9 from *S. japonicum*–infected mice at 0 (NC), 4, 7, 9 and 12 weeks. Data are presented as mean ± SD. **P < 0.01 compared with NC; ^##^ *P* < 0.01 compared with 7 weeks

The function of Th9 cells relies on their ability to secret IL-9. Thus, we measured the expression levels of IL-9 in serum. As demonstrated in Fig. 5, consistent with the generation of Th9 cells, the level of IL-9 increased very quickly in the first four weeks post-infection compared to normal controls, peaked at 7 weeks and decreased gradually thereafter (ANOVA: *F*_(4,20)_ = 29.08, *P* < 0.0001).

## Discussion

Schistosomiasis is a typical chronic infectious disease. Both humoral immunity and cellular immunity participate in the formation and development of the hepatic egg granuloma [21]. Initially, it was thought that this disease was found to elicit preferentially a Th1 response, whereafter provoke an expansion of Th2 response. During the past several years, it has been shown that Th17 and Treg, as well as Tfh cells are involved in the course of *S. japonicum* infection [6–10]. However, the role of Th9 cells in schistosomiasis is unknown. In this study, the *S. japonicum*-infected mouse model was used to observe the dynamic changes of Th9 cells in immunopathogenesis of schistosomiasis. Our findings showed that the proportion of splenic Th9 cells were significantly increased in *S. japonicum*-infected mice compared to normal controls. Th9 cells have been recognized as a predominant source of IL-9 production since Th9 was initially identified as a specific new T cell subset [17, 18]. Subsequently, we found that serum concentrations and liver expression of IL-9 were significantly higher in *S. japonicum*-infected mice than those of in normal controls. PU.1 is a key transcription factor for the differentiation of Th9 cells [22]. Naive CD4^+^ T cells specific deletion of PU.1 had decreased the production of IL-9 in vitro as well as in vivo, even though they were cultured under Th9 conditions [23]. Meanwhile, our data displayed that liver expression of PU.1 had synchronous change tendency with the proportion of Th9 cells and the level of IL-9. Thus, the percentages of Th9 cells, the level of IL-9 and PU.1 all significantly elevated in mice with schistosomiasis, suggesting that the microenvironment induced by *S. japonicum* antigens favors Th9 proliferation and IL-9 secretion.

In recent years, a growing body of evidence suggests that Th9 cells and IL-9 appear to be able to initiate a broad range of immune-mediated inflammatory diseases [19, 24–27]. For example, in the experimental mouse model of hepatic fibrosis due to carbon tetrachloride (CCl4), the percentages of splenic Th9 cells also ascended rapidly and peaked at the 6th week, meanwhile serious hepatic inflammation occurred around this time. Treatment with IL-9-neutralizing antibodies effectively attenuated hepatic inflammation and necrosis [28]. In allergic airway disease, allergic patients have more circulating numbers of Th9 cells than non-allergic control subjects [25]. Neutralization of IL-9 was capable of reducing allergen-induced inflammation [29]. These reports supported a role for Th9 cells in mediating inflammatory response. In this study, we performed histopathological study at different stages of infection to explore the potential relationships between hepatic granulomatous inflammation and Th9 cells. H & E staining showed that the egg granulomas began to form from 4 weeks after infection and reached their volume peak rapidly at 7 weeks. Our immunohistochemical results showed that the expressions of IL-9 and PU.1 were mainly localized around eggs. More importantly, the trends of IL-9 and PU.1 expressions were consistent with the development of egg granulomas. These results demonstrated that Th9 cells and IL-9 were involved in the formation of hepatic egg granulomatous inflammation. As known, eosinophils, macrophages, CD4^+^ T cells, B cells and fibroblasts are the major constituents of egg granulomas, while IL-9 could promote influx and local maturation of eosinophils in parasite-targeted tissues [30, 31], increase the production of IgE from B cells [32] and recruit Th17 cells via CCL-20 [33, 34]. These indicated that Th9 cells may be promoters in the formation of granulomatous inflammation. After 7 weeks post-infection, an anti-inflammatory Th2 response played a dominant role. So pro-inflammatory Th9 cells and egg granulomatous inflammation were decreased.

## Conclusions

To our knowledge, the present study is the first report to provide a preliminary insight into the effects of Th9 cells and IL-9 in the process of murine schistosomiasis. Our data indicated that frequencies of Th9 cells and the level of their specific cytokine were significantly higher in schistosomiasis mice than in NC. Moreover, Th9 cell levels were consistent with the trend of egg granulomatous inflammation in liver, indicating that Th9 cells might be involved in immunopathogenesis in schistosomiasis infection. Further studies are needed to elucidate the detailed roles of Th9 cells in the immunopathogenesis of schistosomiasis.

## References

[1] Wilson MS, Mentink-Kane MM, Pesce JT, Ramalingam TR, Thompson R, Wynn TA (2007). Immunopathology of schistosomiasis. Immunol Cell Biol.

[2] Barsoum RS, Esmat G, El-Baz T (2013). Human schistosomiasis: clinical perspective: review. J Adv Res.

[3] Chuah C, Jones MK, Burke ML, McManus DP, Gobert GN (2014). Cellular and chemokine-mediated regulation in schistosome-induced hepatic pathology. Trends Parasitol.

[4] Mbow M, Larkin BM, Meurs L, Wammes LJ, de Jong SE, Labuda LA (2013). T-helper17 cells are associated with pathologyin human schistosomiasis. J Infect Dis.

[5] Kaplan MH, Whitfield JR, Boros DL, Grusby MJ (1998). Th2 cells are required for the *Schistosoma mansoni* egg-induced granulomatous response. J Immunol.

[6] Wen X, He L, Chi Y, Zhou S, Hoellwarth J, Zhang C, et al. Dynamics of Th17 cells and their role in *Schistosoma japonicum* infection in C57BL/6 mice. PLoS Negl Trop Dis. 2011;5(11):e1399. 10.1371/journal.pntd.0001399 PMC3216943 22102924

[7] Chen X, Yang X, Li Y, Zhu J, Zhou S, Xu Z (2014). Follicularhelper T cells promote liver pathology in mice during *Schistosoma japonicum* infection. PLoS Pathog.

[8] Zhang Y, Jiang Y, Wang Y, Liu H, Shen Y, Yuan Z (2015). Higher frequency of circulating PD-1^high^ CXCR5^+^CD4^+^ Tfh cells in patients with chronic schistosomiasis. Int J Biol Sci.

[9] Zhang Y, Huang D, Gao W, Yan J, Zhou W, Hou X (2015). Lack of IL-17 signaling decreases liver fibrosis in murine schistosomiasis japonica. Int Immunol.

[10] Hesse M, Piccirillo CA, Belkaid Y, Prufer J, Mentink-Kane M, Leusink M (2004). The pathogenesis of schistosomiasisis controlled by cooperating IL-10-producing innate effector and regulatory T cells. J Immunol.

[11] Rutitzky LI, Stadecker MJ (2011). Exacerbated egg-induced immunopathology in murine *Schistosoma mansoni* infection is primarily mediated by IL-17 and restrained by IFN-γ. Eur J Immunol.

[12] Henri S, Chevillard C, Mergani A, Paris P, Gaudart J, Camilla H (2002). Cytokine regulation of periportal fibrosis in humans infected with *Schistosoma mansoni*: IFN-gamma is associated with protection against fibrosis and TNF-alpha with aggravation of disease. J Immunol.

[13] Arnaud V, Li J, Wang Y, Fu X, Mengzhi S, Luo X (2008). Regulatory role of interleukin-10 and interferon-gamma in severe hepatic central and peripheral fibrosis in humans infected with *Schistosoma japonicum*. J Infect Dis.

[14] Kaplan MH (2013). Th9 cells: differentiation and disease. Immunol Rev.

[15] Li P, Spolski R, Liao W, Leonard WJ (2014). Complex interactions of transcription factors in mediating cytokine biology in T cells. Immunol Rev.

[16] Zhao P, Xiao X, Ghobrial RM, Li XC (2013). IL-9 and Th9 cells: progress and challenges. Int Immunol.

[17] Dardalhon V, Awasthi A, Kwon H, Galileos G, Gao W, Sobel RA (2008). IL-4 inhibits TGF-beta-induced Foxp3+ T cells and, together with TGF-beta, generates IL-9+ IL-10+ Foxp3(−) effector T cells. Nat Immunol.

[18] Veldhoen M, Uyttenhove C, van Snick J, Helmby H, Westendorf A, Buer J (2008). Transforming growth factor-beta ‘reprograms’ the differentiation of T helper 2 cells and promotes an interleukin9-producing subset. Nat Immunol.

[19] Li H, Nourbakhsh B, Cullimore M, Zhang GX, Rostami A (2011). IL-9 is important for T-cell activation and differentiation in autoimmune inflammation of the central nervous system. Eur J Immunol.

[20] Tuxun T, Apaer S, Ma HZ, Zhang H, Aierken A, Lin RY (2015). The potential role of Th9 cell related cytokine and transcription factors in patients with hepatic alveolar echinococcosis. J Immunol Res.

[21] Pearce EJ, Macdonald AS (2002). The immunobiology of schistosomiasis. Nat Rev Immunol.

[22] Goswami R, Kaplan MH (2012). Gcn5 is required for PU.1-dependent IL-9 induction in Th9 cells. J Immunol.

[23] Chang HC, Sehra S, Goswami R, Yao W, Yu Q, Stritesky GL (2010). The transcription factor PU.1 is required for the development of IL-9-producing T cells and allergic inflammation. Nat Immunol.

[24] Tan C, Aziz MK, Lovaas JD, Vistica BP, Shi G, Wawrousek EF (2010). Antigen-specific Th9 cells exhibit uniqueness in their kinetics of cytokine production and short retention at the inflammatory site. J Immunol.

[25] Jones CP, Gregory LG, Causton B, Campbell GA, Lloyd CM (2012). Activin a and TGF-beta promote T(H)9 cell-mediated pulmonary allergic pathology. J Allergy Clin Immunol.

[26] Sehra S, Yao W, Nguyen ET, Glosson-Byers NL, Akhtar N, Zhou B (2015). TH9 cells are required for tissue mast cell accumulation during allergic inflammation. J Allergy Clin Immunol.

[27] Ciccia F, Guggino G, Ferrante A, Cipriani P, Giacomelli R, Triolo G (2016). Interleukin-9 and T helper type 9 cells in rheumatic diseases. Clin Exp Immunol.

[28] Qin SY, Lu DH, Guo XY, Luo W, Hu BL, Huang XL (2016). A deleterious role for Th9/IL-9 in hepatic fibrogenesis. Sci Rep.

[29] Kearley J, Erjefalt JS, Andersson C, Benjamin E, Jones CP, Robichaud A (2011). IL-9 governs allergen-induced mast cell numbers in the lung and chronic remodeling of the airways. Am J Respir Crit Care Med.

[30] Louahed J, Zhou Y, Maloy WL, Rani PU, Weiss C, Tomer Y (2001). Interleukin 9 promotes influx and local maturation of eosinophils. Blood.

[31] Licona-Limon P, Henao-Mejia J, Temann AU, Gagliani N, Licona-Limon I, Ishigame H (2013). Th9 cells drive host immunity against gastrointestinal worm infection. Immunity.

[32] Dugas B, Renauld JC, Pène J, Bonnefoy JY, Peti-Frère C, Braquet P (1993). Interleukin-9 potentiates the interleukin-4-induced immunoglobulin (IgG, IgM and IgE) production by normal human B lymphocytes. Eur J Immunol.

[33] Nowak EC, Weaver CT, Turner H, Begum-Haque S, Becher B, Schreiner B (2009). IL-9 as a mediator of Th17-driven inflammatory disease. J Exp Med.

[34] Zhou Y, Sonobe Y, Akahori T, Jin S, Kawanokuchi J, Noda M (2011). IL-9 promotes Th17 cell migration into the central nervous system *via* CC chemokine ligand-20 produced by astrocytes. J Immunol.

